# *“Making a difference”* – Medical students’ opportunities for transformational change in health care and learning through quality improvement projects

**DOI:** 10.1186/s12909-016-0694-1

**Published:** 2016-07-11

**Authors:** Anne-Marie Bergh, Martin Bac, Jannie Hugo, John Sandars

**Affiliations:** South African MRC Unit for Maternal and Infant Health Care Strategies, University of Pretoria, Private Bag X323, Pretoria, Arcadia 0007 South Africa; Department of Family Medicine, University of Pretoria, Pretoria, South Africa; Academic Unit of Medical Education, University of Sheffield, Sheffield, UK

**Keywords:** Quality improvement, Transformative learning, District health rotation, Medical education, Self-authorship

## Abstract

**Background:**

Quality improvement is increasingly becoming an essential aspect of the medical curriculum, with the intention of improving the health care system to provide better health care. The aim of this study was to explore undergraduate medical students’ experiences of their involvement in quality improvement projects during a district health rotation.

**Methods:**

Student group reports from rotations in learning centres of the University of Pretoria in Mpumalanga Province, South Africa were analysed for the period 2012 to 2015. Interviews were conducted with health care providers at four learning centres in 2013.

**Results:**

Three main themes were identified: (1) ‘Situated learning’, describing students’ exposure to the discrepancies between ideal and reality in a real-life situation and how they learned to deal with complex situations, individually and as student group; (2) ‘Facing dilemmas’, describing how students were challenged about the non-ideal reality; (3) ‘Making a difference’, describing the impact of the students’ projects, with greater understanding of themselves and others through working in teams but also making a change in the health care system.

**Conclusion:**

Quality improvement projects can provide an opportunity for both the transformation of health care and for transformative learning, with individual and ‘collective’ self-authorship.

## Background

Quality improvement (QI) is fast becoming an essential component of the practical curriculum in undergraduate medical education, with the intention of improving health care through making changes to the health care system [1]. However, there are several approaches to learning QI in the undergraduate curriculum. Wong et al. [2] highlight the important difference between the teaching of QI concepts and methods in formal curriculum activities compared with the immersion of students in participatory activities that occur in real-world health settings. This distinction is important since in this paper we propose that participation in QI projects has the potential to not only transform health care but also lead to transformative learning in the students.

Transformative learning occurs when the worldview of a learner is challenged by an experience [3]. These experiences create a “disorientating dilemma” [4] and the complexity of real-world contexts provides numerous experiences to stimulate transformative learning. This process has long been recognised as an outcome of service learning, when students participate in community projects [5]. QI projects are a specific type of service learning. An important aspect of transformative learning is the personal growth and development of both individuals and groups but we have identified no previous studies that describe this aspect for medical students participating in QI projects. Previous research has had a focus on the acquisition of specific competences related to QI [6].

Self-authorship provides a useful framework to understand personal growth and development in undergraduate medical students. The concept of self-authorship was proposed by Baxter-Magolda [7] and is based on her longitudinal study of a cohort of individuals from late adolescence to early adulthood. She identified personal growth and development along three inter-related dimensions: cognitive, intrapersonal and interpersonal. The cognitive dimension describes how an individual perceives the certainty of knowledge, with a movement over time towards a recognition that the real world is complex and that there are different perspectives. The intrapersonal dimension is related to the development of an integrated identity over time, with the recognition that identity and beliefs are not fixed. The inter-personal dimension concerns relationships, with an appreciation of diversity. Personal growth and development occurs when there are “crossroad” experiences and these can be stimulated through service learning [8]. We have identified no previous studies of self-authorship in undergraduate medical students participating in QI projects.

An essential aspect of both transformative learning and self-authorship is situated learning, with learning occurring through participation and activities within an authentic context [9]. This perspective highlights that learning occurs through action and reflection on actions, with challenge of current worldviews. Billett [10] has emphasised the importance of learning within groups, with collective reflection to widen the challenge to individual worldviews.

The medical curriculum of the University of Pretoria in South Africa includes a 7-week district health rotation in the final (fifth or sixth) year of the undergraduate programme. Annually between 130 and 140 students are placed at a number of clinical learning centres linked to a district, regional or tertiary hospital in Mpumalanga Province, South Africa. In the period 2012 to 2015 students were involved in longitudinal QI projects focused on the Mother and Baby Friendly Initiative (MBFI), a priority of the national Department of Health [11] aimed at protecting, promoting and supporting breastfeeding [12]. Lecturers provided oral and written orientation to the QI projects at the beginning of each rotation and onsite mentors provided moral and technical support throughout the rotation. A group report was submitted at the end of a rotation, on which students received feedback from the lecturers. Each student group had to come up with recommendations on which subsequent groups were to follow up. A QI spiral was created through the monitoring of MBFI practices over a period of 4 years.

The aim of this study was to explore undergraduate medical students’ experiences of their involvement in QI projects during a district health rotation. Adopting a longitudinal qualitative approach enabled us to get a sense of the more lasting effect medical students’ continuous involvement in the MBFI programme could have. However, we were not only interested in the potential of QI projects to transform health care, but also how these projects could lead to transformative learning in the students, especially from a self-authorship perspective.

## Methods

The study protocol was approved by the Research Ethics Committee of the University of Pretoria (S160/2009) and the Mpumalanga Research and Ethics Committee.

The study was conducted in Mpumalanga Province with all student groups (except for one) in all district health rotations in 2012. This was followed up in 2013 to 2015 with the last rotation of each year devoted to the MBFI as QI topic. In total 229 students were involved, spread across nine clinical learning centres and over nine different rotation periods.

### Data collection

For 2012 two sources of data were available from the students:

- Student QI reports (*n* = 34) (collective documents produced per group per site)
- Other documents produced and/or collected during their rotation (e.g. posters, leaflets, PowerPoint presentations, policy documents, minutes of meetings).

In order to get a more longitudinal perspective and for further triangulation, two data collection activities were undertaken between 2013 and 2015:

- Focus group interviews conducted with health care providers at four learning centres in 2013 and one individual interview with a nursing manager actively engaged with students, but who was not available for participation in a focus group.
- Student QI reports (*n* = 22), presentations and related documents for the last rotation of each of the years 2013 to 2015.

### Data analysis

The data were analysed inductively over the 4-year period of data collection. The analysis approach incorporated some elements of grounded theory. Two authors (A-MB, MB) initially immersed themselves in the different texts and did independent analyses. At their regular meetings, which resembled an iterative process, interpretations were discussed and compared and chronological developments over time were traced. The themes emerging from the students’ documents were also compared with the relevant themes identified in the health-provider interviews. In the course of time the analysis, which took on a form of constant comparison, moved from the initial (open) coding to axial coding of the data into categories and themes.

Direct quotations reported in the findings below are marked with the following codes: HA, HB, HC, etc. = individual hospitals; FG = focus group; II – individual interview; SR = student report; R = rotation.

## Results

Three main themes were identified from the analysis. *Situated learning* describes students’ exposure to the ideal-reality discrepancies in a real-life situation. *Facing dilemmas* includes the numerous occasions in which students were challenged about the non-ideal reality and were forced to sometimes make unpopular choices. The third theme relates to the outcome of students’ QI projects, which could be summarised as *“Making a difference”*. Table 1 gives a summary of the themes, sub-themes and categories.

**Table 1:** Main themes, sub-themes and categories

Themes	Sub-themes	Categories
1. Situated learning	Levels of situated learning	• Broader environment/situation
		• Hospital as a place of learning
		• Study area to master
	Nature of learning	• Individual learning
		• Collective learning
2. Facing dilemmas	Leadership	• In hospital
		• In student group
	Commitment	• In hospital
		• In student group
	Continuity	• In hospital
		• In student group
3. Making a difference	Collaborations	• Intra-group collaboration
		• Inter-group collaboration
	Making a difference in a rotation	• Feeling of making a difference
		• Making a short term difference
	Making a long-term difference	• Impact on the health system
		• Difficult to measure impact

### “*We took a while to understand the climate of the hospital” –* situated learning

Situated learning was made visible through student activities (training, development of materials, updating of policy). The complexity of the QI project was central to learning as students *“soon realised that there is a lot of aspects of our project that we don’t have control over”.* (HF SR R6 2015) Two situated-learning sub-themes were identified: levels of situated learning, and the nature of individual and collective learning.

### Levels of situated learning

There were three levels of situated learning: the broader environment or situation, the hospital as place of learning, and the area of study to master.

### The broader environment or situation

The district health rotations were the first time students were placed in a new environment as a group and they had to work across different disciplines to conduct their QI project. It was also the first time that most students had been exposed to rural health and issues related to the public health system in resource-constrained settings.

*“We feel we have made a concerted effort to fully understand the dynamics surrounding the MBFI/BFHI both within the hospital as well its reaches beyond the gates of the hospital … there a[re] plenty of issues at hand, and to try and rectify them all at once seems incredibly ambitious … a sensible and realistic approach seems to [be] the only solution …”* (HA SR R6 2013)

### The hospital as a place of learning

Some hospitals were well established but others had a high turnover of managers, which affected the organisation of a QI project and students’ efforts to obtain staff cooperation.

*“Getting information from the healthcare workers was a battle. People there didn’t seem to be willing to talk to us … Some even showed emotional components.”* (HC SR R6 2013)

### The area of study to master

Students did not have control over their choice of topic but they had room to steer their focus according to need and interest [13].

*“During our time at Hospital A we learnt a lot about the practices in place after childbirth. We all know the facts, and the information regarding skin-to-skin practices and their benefit to mother and baby alike. We can all educate sisters and patients on such benefits, but probably the most vital thing we learnt is that it just is not that simple.”* (HA SR R6 2013)

*“By doing this QIP, not only have we discovered the functioning of the MBFI at Hospital D, but we have gained a clear understanding of what it actually is and why it is so important to implement the 10 steps that the WHO and UNICEF have implemented.”* (HD SR R6 2014)

### Nature of learning (individual and collective)

Students had to become familiar with a specific public health topic, in this instance learning about infant feeding through the MBFI, and they also had to learn about the organisation of programmes and the importance of human relations in health care delivery. This resulted in individual and collective learning.

*“We discussed this problem with the nursing staff and midwives of labour ward, the doctor in charge of labour ward, our mentor and the medical manager. Together it was decided that early initiation of breastfeeding and the barriers preventing implementation is an essential topic to address in the labour ward.”* (HF SR R3 2014)

### *“There are a number of struggles”* – facing dilemmas

Students faced different dilemmas in the course of executing their QI projects in which their relationship skills were tested to the full. In their group, students had to deal with different issues affecting the functioning of the group, such as the short time frame for completing their project. The main dilemmas are described in terms of three overlapping subthemes on leadership, commitment and continuity, from a students’ perspective and a hospital/learning perspective.

### Leadership

Students were confronted with different leadership styles that spilled over in the management of boundaries between different units in poorer functioning hospitals (e.g. antenatal, labour, postnatal wards) and in interprofessional relationships (e.g. mistrust between nurses and dieticians).

There were two leadership areas: in the hospital, especially through activities in the breastfeeding committee, and in the student rotation group. In some sites students took the lead in addressing the dilemma of absent leaders in MBFI. One group, for example, adopted a soft approach in getting a breastfeeding committee established:

*“We then decided to identify the key role players … based on their involvement, their willingness to interact with us and their level of knowledge about the hospital and practices … we allowed them to feel like the committee was their ‘baby’ rather than an external influence upon them. This means they are more motivated to do the work.”* (HB SR R6 2014)

Dynamics and leadership within the student group were also mentioned. In some groups the selection of a leader “*occurred through a natural process”* that “*needed no structured consultation”* (HH SR R6 2013), but *“inherently strong leadership instincts of all … group members”* (HH SR R6 2013) were not helpful.

### Commitment

Students learned about the presence and absence of passion and commitment among health workers and about their own commitment. They also observed *“lack of motivation”* (HA SR R6 2012), *“a general lack of interest”* (HG SR R6 2013) and lack of “*input, enthusiasm and support from crucial parties”* (HA SR R6 2013) with *”no one … willing to take a step forward.”* (HC SR R6 2013) Despite the challenges, students were committed to make a success of their project:

*“We were very passionate about the topic before us and therefore we were extremely motivated to make it a success. We worked very hard and did extensive research.”* (HA SR R4 2015)

### Continuity

Taking the MBFI forward was hampered by a lack of continuity of staff and the fact that each student group was only present on site for five weeks. Particularly crucial was “*a change of staff members, especially the leaders of the committee”* (HC SR R6 2013) and in one hospital the departure of *“a doctor who was very committed to our committee.”* (HG FG 2013)

### *“Making a difference”* – outcomes of the quality improvement projects

A large number of student reports referred to the fact of *“making a difference”*. The three main making-a-difference subthemes are collaboration, student perceptions of making a difference in a rotation, and making a long-term difference.

### Collaborations

Collaboration and teamwork was a major student learning experience, with students exposed to different types of collaborations that could broadly be described as intra-group collaboration between students and inter-group collaboration between students and hospital staff.

*“We worked together as a dynamic and integrated unit and equally shared responsibilities amongst the group members. In addition we collaborated well with the hospital staff and functioned in unison.”* (HA SR R6 2014)

Students learned to understand the functioning of their team, *“each other’s strengths and weaknesses, preferences and aversions”*, they “*developed increased confidence in our own abilities”* and learned how to deal with conflict through “*effective negotiation and collaboration”* that left *“the group unity … unscathed.”* (HH SR R6 2013)

*“We all had to overcome some challenges while working in our team. Within our group of medical students we have 5 strong personalities & we sometimes disagreed on how to approach a specific problem. For all of us it was a good learning experience in conflict management. We also had times of fun & bonding, especially during data capturing in Town X’s Wimpy [diner].”* (HF SR R6 2015)

Students appreciated that teamwork “*in implementing any management plans and involving all the variety of departments and staff members will play a big part in the operational success of the project”.* (HH SR R1 2012) Students had to cooperate closely with staff members involved in the breastfeeding committee and could complement their work to obtain a better picture of the real situation on the ground:

*“We became part of the team as the men (and women) on the ground … The other role we played was to evaluate the real circumstances and how possible it is to implement all the policies in the real situations in the wards.”* (HF SR R6 2013)

Students learned the hard way on how to overcome unintended consequences of actions.

*“We … had to navigate issues that they [local health workers] were sensitive about. Unfortunately sometimes we inadvertently offended them and had to explain ourselves. But we soon realised that without all our team members we wouldn’t have been able to complete our project. The workload simply would have been too much and we needed the insight and assistance of our local team members.”* (HF SR R6 2015)

### Making a difference in a rotation

Making a difference in a rotation related to visible short-term outcomes and was also reflected in students’ hopes for the continuation of short-term achievements:

*“We saw some of our suggestions immediately implemented after our presentation. This made us feel great that we could effect change. However, these achievements should not cause us to sit back and relax. As the QI cycle shows, it is a continuous process of reflection and identifying problems and looking for solutions to these problems.”* (HH SR R6 2015)

### Making a long-term difference

Generally, the long-term impact of the student QI projects in terms of changes in the health system and provider behaviour could not be observed in individual student rotations. Some student groups in the later rotations did observe three types of changes: positive changes, changes hampered by fundamental challenges, or no change at all, as is illustrated by the following comments:

*“Hospital H was formally recognized as a mother-baby friendly hospital in 2012 … The breastfeeding committee expressed a great amount of gratitude … the majority of the input was made in the relevant wards, where practices were changed significantly. They [breastfeeding committee] … received extremely helpful recommendations from this [2013] group of students.”* (HH SR R6 2013)

*“In summary the previous groups made many valuable contributions to improving the problems … But the core of the problem is still unresolved. It can be traced back to two main obstacles: a leader for the committee and training of the staff. These two main problems cannot be changed by students, unfortunately.”* (HA SR R6 2012)

*“Our findings were not encouraging at all. We realized that these projects are done, reports are written, recommendations are made, but not much is changed.”* (HB SR R6 2013)

Health care providers in the health system whom we interviewed could relate to changes in which students played a crucial role as catalysts [13] – *“They realise they follow on to each other and try to complete what others could not and things like that, that is an important thing they are doing.”* (HD FG 2013)

A strong observation from health care providers was the role of the students to raise awareness: “*The quality improvement definitely opened people’s eyes again”* (HD FG 2013), and *“You know, someone from outside helps a lot.”* (HG FG 2013)

Specifically mentioned as an eye-opening role of students was their ability to raise awareness among members of the medical profession, who had not been much involved in the MBFI before:

*“If it wasn’t for them, still nothing would have happened … Really I think they made a difference by being here, just to create awareness, especially [with] … the other doctors as well, because it is difficult for us to reach them.”* (HG FG 2013)

## Discussion

Our findings illustrate how QI projects by undergraduate medical students can provide an important opportunity for both the transformation of health care and for transformative learning, with individual and ‘collective’ self-authorship. An essential aspect of the learning is the variety of transformative experiences stimulated by immersion within a real-world context. This is in line with a finding by Couper [14] on the personal growth of South African undergraduate medical students during a rural health elective.

The identified themes and quotations vividly illustrate personal growth and development as self-authorship across all three dimensions: cognitive, intrapersonal and interpersonal. Existing world views were challenged by “crossroad” experiences [8] that were stimulated by the wide range of different real-world situations that the students faced. The range of situations is closely inter-related and they arise from both the implementation of the QI project and the working as a group.

**Fig. 1:**
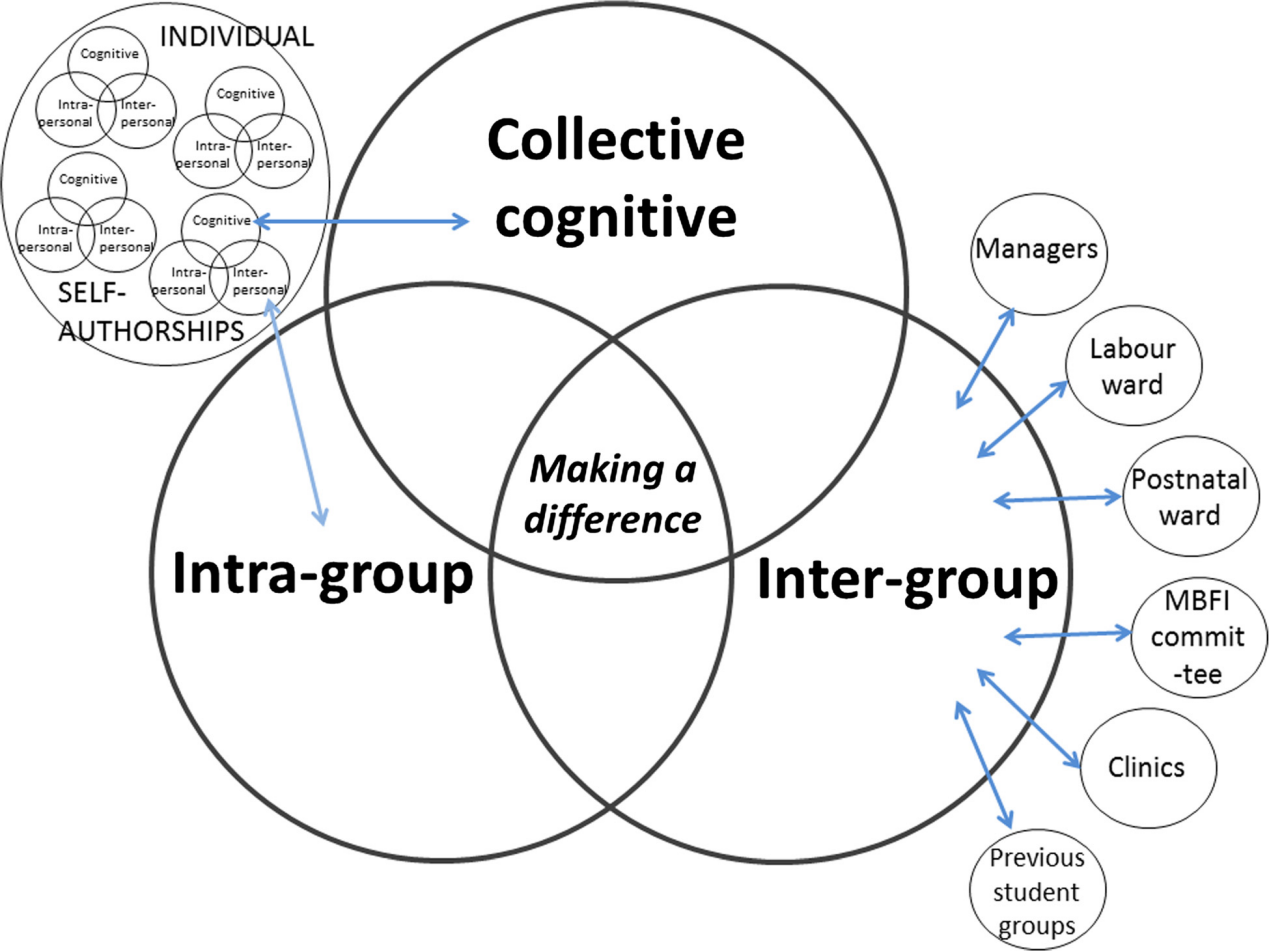
‘Collective’ self-authorship in context

We argue that during the QI project a ‘collective’ self-authorship occurs because the students have an intense period of engagement with their group but also with the local health care context. Our conceptualisation of ‘collective’ self-authorship is depicted in Fig. 1. The development of individual self-authorship and ‘collective’ self-authorship occur concurrently and ‘collective’ is especially related to the interpersonal component of individual self-authorship. Just as with individual self-authorship, ‘collective’ authorship also consists of three overlapping dimensions – the group developed or matured cognitively as individuals through their group activities, they reflected on their own position as a group and they established relationships with other groups as part of their learning partnerships. ‘Collective’ self-authorship could be summarised by students’ reflections of making a difference. The category of mastering of subject matter is closely linked to the students’ cognitive development. An example of intra-group matters relates to the way in which intra-group conflicts were resolved. The inter-group tensions between dieticians and nurses were prominent at some sites and students had to learn how to deal with situations where they found themselves in the proverbial line of fire.

The group engagement during the QI project establishes a collective learning partnership that is a key educational process for self-authorship. The learning partnership model proposes three essential, and interrelated, aspects of any educational experience with a focus on self-authorship [15], which is also applicable to ‘collective’ self-authorship:

- Knowledge is complex: learners have to experience situations in which ways of knowing, or knowledge, have multiple interpretations that depend not only on information and facts, but also on the beliefs and values that influence its interpretation. These situations require exposure to real-life challenges and lead development in the cognitive dimension.
- The self is central to knowledge construction: learners have to take on responsibility for learning, with a willingness to challenge their existing worldview, including their beliefs about both themselves and others. This process leads to the development of the intrapersonal dimension of self-authorship.
- Authority and expertise is shared through interaction with peers: learners begin to fully appreciate that effective learning is a mutual process, with sharing and tolerance of different perspectives. This process primarily develops the interpersonal dimension of self-authorship.

The complexity of the three levels of the learning environment that we described (the broader environment or situation; the hospital as place of learning; and the area of study to master) exposed students to different ways of knowing and made them realise that reality could imply multiple interpretations based not only on factual knowledge, but also on varying values and beliefs that challenged their existing worldviews [16]. In the QI project, theoretical didactic learning is replaced by situated, experiential learning. McMillan argues that “learning happens first in the social and then in the individual plane, i.e. [learning] is an inherently social practice” [17]. This was reflected in the different collaborative relationships students had to establish in the course of their QI project.

The two main conditions inherent in the learning partnerships model are support and challenge [15]. These conditions require learners to be challenged within a supportive relationship and environment, thereby facilitating the learner and the group to progress across the developmental phases of individual and ‘collective’ self-authorship respectively. In our study there was continuous intra-group support among the students and inter-group support by lecturers and onsite mentors and health professionals.

Our study had a number of limitations. The impact of the students’ QI projects could not be measured quantitatively because of the complex health context in which they were situated while doing their projects [13]. The QI project was one part of the broader district health rotation and the evaluation of this programme was not the aim of this study. Furthermore, the dimensions of individual and collective self-authorships could not be explored in sufficient depth because the study was not designed with this framework in mind. We therefore did not have access to individual students’ reflections or major-change stories to support our analysis. In order to be able to build on our initial interpretation of ‘collective’ self-authorship, more studies would be needed to explore our initial conceptualisation in more depth.

## Conclusion

In this paper we attempted to illustrate how QI projects served as vehicle for individual and especially ‘collective’ self-authorship. Students were confronted with a different approach to what they had been exposed to before and they were able to realise how things worked in practice. District and rural health rotations provide medical students with real-life opportunities for focused QI projects at hospital and community level. This is where students could experience transformative learning through taking responsibility and contribute to making a difference in transforming public health facilities. Of particular importance is the inter-group relationship dimension with its potential to lead to “… increasing appreciation of different beliefs, values and attributes between individuals and groups, with greater awareness of cultural competence, team working and social accountability” [16]. Medical educators should create opportunities and conditions for students to be involved in real-life QI as part of their training.

## Abbreviations

FG, focus group; HA, HB, HC, etc., individual hospitals; II, individual interview; MBFI, mother and baby friendly initiative; QI, quality improvement; SR, student report; R, rotation.
